# YeastMate: neural network-assisted segmentation of mating and budding events in *Saccharomyces cerevisiae*

**DOI:** 10.1093/bioinformatics/btac107

**Published:** 2022-02-18

**Authors:** David Bunk, Julian Moriasy, Felix Thoma, Christopher Jakubke, Christof Osman, David Hörl

**Affiliations:** Faculty of Biology, Ludwig-Maximilians-Universität München, 82152 Planegg-Martinsried, Germany; Faculty of Biology, Ludwig-Maximilians-Universität München, 82152 Planegg-Martinsried, Germany; Faculty of Biology, Ludwig-Maximilians-Universität München, 82152 Planegg-Martinsried, Germany; Faculty of Biology, Ludwig-Maximilians-Universität München, 82152 Planegg-Martinsried, Germany; Faculty of Biology, Ludwig-Maximilians-Universität München, 82152 Planegg-Martinsried, Germany; Faculty of Biology, Ludwig-Maximilians-Universität München, 82152 Planegg-Martinsried, Germany

## Abstract

**Summary:**

Here, we introduce *YeastMate*, a user-friendly deep learning-based application for automated detection and segmentation of *Saccharomyces cerevisiae* cells and their mating and budding events in microscopy images. We build upon Mask R-CNN with a custom segmentation head for the subclassification of mother and daughter cells during lifecycle transitions. *YeastMate* can be used directly as a Python library or through a standalone application with a graphical user interface (GUI) and a Fiji plugin as easy-to-use frontends.

**Availability and implementation:**

The source code for YeastMate is freely available at https://github.com/hoerlteam/YeastMate under the MIT license. We offer installers for our software stack for Windows, macOS and Linux. A detailed user guide is available at https://yeastmate.readthedocs.io.

**Supplementary information:**

Supplementary data are available at *Bioinformatics* online.

## 1 Introduction

An important experimental approach when working with the budding yeast *Saccharomyces cerevisiae* is to examine the effects of mutations on morphological features or protein localization either by brightfield or fluorescent microscopy (Ohya *et al.*, 2015). Such experiments can also be performed in a systematic manner by combining automated microscopy with strain libraries comprising thousands of yeast strains that carry gene deletions or express fluorescently tagged proteins (Giaever *et al.*, 2002; Huh *et al.*, 2003; Weill *et al.*, 2018), which, however, calls for robust automated image analysis pipelines. In recent years, tools based on convolutional neural networks (CNNs) have become state-of-the-art for many tasks in biomedical image analysis (von Chamier *et al.*, 2019), including segmentation of individual *S.cerevisiae* cells (Dietler *et al.*, 2020; Lu *et al.*, 2019; Salem *et al.*, 2021). Many experimental strategies facilitated by the yeast system also make use of specific transitions in the yeast lifecycle like budding and mating to study things like organelle inheritance and mitochondrial quality control (Jakubke *et al.*, 2021; Rafelski *et al.*, 2012). Detection of matings and buddings is often done by hand or through dedicated postprocessing routines on the output of a single-cell segmentation tool (e.g. tracking of cells in a time series of images), highlighting the need for easy-to-use end-to-end solutions for these more complex tasks.

Here, we present *YeastMate*, a novel deep learning-based tool for end-to-end segmentation of single cells and detection of transitions in the lifecycle of *S.cerevisiae* in single transmitted light images. YeastMate performs three tasks: instance segmentation of single cells, object detection of zygotes and budding events and automatic assignment of mother and daughter cells involved in a mating or budding event. The detection backend is based on Mask R-CNN (He *et al.*, 2017) and is complemented by a user-friendly frontend using modern web technologies as well as a Fiji plugin. In the task of detecting mating and budding events, we achieve accuracies comparable to manual human reannotation and we can also perform single-cell segmentation robustly across various datasets. YeastMate is already being used in ongoing research in our lab (Jakubke *et al.*, 2021). In addition to the software, we also provide a new dataset of images with manually annotated cells and mating and budding events.

## 2 Materials and methods

*CNN architecture and training*: Our network architecture builds upon Mask R-CNN with a modified mask segmentation head producing multiclass semantic segmentations (Supplementary Fig. S1) for each detected object. Instead of individually detecting mother and daughter cells, we first detect the whole mating and budding events as well as single cells. In a postprocessing step, we resolve the roles of the cells involved in budding or mating events based on the multiclass segmentation masks (Supplementary Fig. S2). We used 80% of our images for training our network and cross-validation and 20% as a hold-out test set for the final performance assessment.

**Fig. 1.**
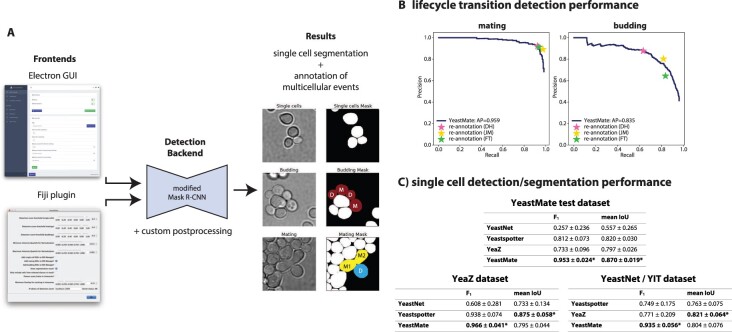
(**A**) Main components and output of *YeastMate*: We perform instance segmentation of single cells and detection of lifecycle transitions using a modified Mask R-CNN (middle), which can either be used directly from Python code or via two GUI frontends (left) to provide instance segmentation of single cells as well as detection of budding events and mating events with identification of the mother (M) and daughter (D) cells involved in the event (right). (**B**) Precision–recall (PR) curves of YeastMate performance for detection of mating and budding events in our test dataset. Human precision and recall during reannotation by three different authors are plotted as colored stars. (**C**) Single-cell detection performance (*F*_1_ score) and segmentation performance (mean intersection-over-union of true positive detections) of YeastNet, YeastSpotter, YeaZ and YeastMate on our own test dataset as well as datasets from Dietler *et al.* (2020) and Salem *et al.* (2021). The values in the table are mean ± standard deviation across all images in a dataset

*Software architecture*: YeastMate is implemented in a modular way: the detection backend can be used as a Python library but also runs as a webservice to provide its capabilities to client applications. As clients, we provide a Fiji plugin that directly interfaces with the server via HTTP requests as well as a standalone GUI Desktop application (Fig. 1 and Supplementary Figs S3–S5). We provide the whole YeastMate stack as a single installable file for use on a local workstation.

For more details, please refer to the Supplementary Methods.

## 3 Results

*Dataset*: For training YeastMate, we collected 147 brightfield and differential interference contrast (DIC) images of *S.cerevisiae* acquired on two different microscopes at various imaging conditions and generated curated single-cell segmentation masks and mating and budding annotations. In total, our data contain 17 058 individual cells, 3615 buddings and 2380 zygotes (Supplementary Table S1).

*Object detection performance*: Our network achieves a mean average precision (mAP) of 0.878 as well as favourable APs for the individual classes of objects when applied to our test set (Fig. 1B and Supplementary Fig. S6). We also assessed interhuman reproducibility by comparing the annotations for mating and budding events of four different annotators (with the most experienced one chosen as the reference). On our test dataset, mean interhuman precision and recall are (0.908, 0.946) for mating events and (0.774, 0.763) for budding events. These values lie close to the PR-curves of our network, indicating that YeastMate can achieve object detection performance comparable to manual human annotation.

*Single-cell segmentation performance*: YeastMate also compares favourably to existing CNN-based solutions in single-cell segmentation, showing consistent performance not only on our own test dataset, but also two publicly available datasets (Fig. 1C, Supplementary Results, Supplementary Fig. S7 and Table S2). Based on the robust Mask R-CNN architecture, YeastMate achieves the highest single-cell detection performance of all tools as well as competitive results in segmentation.

## 4 Conclusion

With YeastMate, we introduce an easy-to-use application to not only perform single-cell segmentation in images of *S.cerevisiae* with high robustness across datasets, but also detect transitions in the cell cycle in single images with accuracies comparable to human annotation. YeastMate is implemented in a modular way and can be run on local workstations but the detection server can also be run on a remote compute server. Additionally, we provide two user-friendly frontends to make the tool available without the need to write code. We can envision YeastMate to be expanded to detect other life cycle states, such as meiotic asci, similar to existing work in *Arabidopsis* *thaliana* (Lim *et al.*, 2020). YeastMate can provide a considerable improvement to high-throughput studies of yeast, facilitating not only the automated analysis of large image datasets of single cells, but also enabling the study the complex interplay of cellular components during both sexual and asexual reproduction of *S.cerevisiae*.

## Supplementary Material

btac107_Supplementary_Data

## Data Availability

The data underlying this article are available in Open Science Framework (OSF), at https://doi.org/10.17605/osf.io/287fr
